# Multimodal treatment for high-risk prostate cancer with high-dose intensity-modulated radiation therapy preceded or not by radical prostatectomy, concurrent intensified-dose docetaxel and long-term androgen deprivation therapy: results of a prospective phase II trial

**DOI:** 10.1186/1748-717X-9-24

**Published:** 2014-01-14

**Authors:** Andrea Guttilla, Roberto Bortolus, Gianluca Giannarini, Pirus Ghadjar, Fabio Zattoni, Michele Gnech, Vito Palumbo, Francesca Valent, Antonio Garbeglio, Filiberto Zattoni

**Affiliations:** 1 Department of Oncological, Surgical and Gastroenterological Sciences, Urology Clinic, University of Padua, Padua 35128, Italy; 2 Department of Radiation Oncology, National Cancer Institute Aviano, Aviano, PN, Italy; 3 Department of Urology, University of Bern, Inselspital, Bern, Switzerland; 4 Department of Radiation Oncology, University of Bern, Inselspital, Bern, Switzerland; 5 Epidemiological Service, Direzione Centrale Salute, Integrazione Sociosanitaria e Politiche Sociali, Regione Autonoma Friuli Venezia Giulia, Udine, Italy; 6 Department of General Surgery, Division of Urology, Azienda Ospedaliera “Santa Maria degli Angeli”, Pordenone, Italy

**Keywords:** Prostatic neoplasms, Radiotherapy, Intensity-modulated, Prostatectomy, Androgen deprivation therapy, Chemotherapy, Docetaxel, Toxicity, Clinical trial, Phase II

## Abstract

**Background:**

The optimal management of high-risk prostate cancer remains uncertain. In this study we assessed the safety and efficacy of a novel multimodal treatment paradigm for high-risk prostate cancer.

**Methods:**

This was a prospective phase II trial including 35 patients with newly diagnosed high-risk localized or locally advanced prostate cancer treated with high-dose intensity-modulated radiation therapy preceded or not by radical prostatectomy, concurrent intensified-dose docetaxel-based chemotherapy and long-term androgen deprivation therapy. Primary endpoint was acute and late toxicity evaluated with the Common Terminology Criteria for Adverse Events version 3.0. Secondary endpoint was biochemical and clinical recurrence-free survival explored with the Kaplan-Meier method.

**Results:**

Acute gastro-intestinal and genito-urinary toxicity was grade 2 in 23% and 20% of patients, and grade 3 in 9% and 3% of patients, respectively. Acute blood/bone marrow toxicity was grade 2 in 20% of patients. No acute grade ≥4 toxicity was observed. Late gastro-intestinal and genito-urinary toxicity was grade 2 in 9% of patients each. No late grade ≥3 toxicity was observed. Median follow-up was 63 months (interquartile range 31–79). Actuarial 5-year biochemical and clinical recurrence-free survival rate was 55% (95% confidence interval, 35-75%) and 70% (95% confidence interval, 52-88%), respectively.

**Conclusions:**

In our phase II trial testing a novel multimodal treatment paradigm for high-risk prostate cancer, toxicity was acceptably low and mid-term oncological outcome was good. This treatment paradigm, thus, may warrant further evaluation in phase III randomized trials.

## Background

Depending on the definition, high-risk localized or locally advanced prostate cancer (PCa) accounts for 15-40% of newly diagnosed cases [1,2]. Despite advances in patient selection and primary/adjuvant therapies, disease recurrence remains substantial, affecting >50% of patients within 10 years after treatment [3,4].

In the absence of adequately conducted randomized trials, optimal treatment remains controversial. External beam radiation therapy (EBRT) with long-term androgen deprivation therapy (ADT) has long been considered the standard of care over other modalities [5,6]. The survival benefit of long-term ADT combined with EBRT vs. EBRT alone has recently been confirmed in a meta-analysis [7]. Over the past decade, however, there has been a surge in the utilization of radical prostatectomy (RP) as primary treatment in selected patients, followed by EBRT in case of adverse pathological features [5,6]. It is currently accepted that a combined modality-regimen should be pursued in clinical trials, although sequence, timing and intensity of the single treatments are still under scrutiny [8].

Docetaxel is an active agent in the treatment of PCa but, until recently, its role has been limited to patients with metastatic castration-resistant disease [9,10]. However, in order to treat micrometastatic disease, an earlier use of chemotherapy within the curative treatment setting may be beneficial. Docetaxel is also known as a radiosensitizer, and has been shown to improve local control in combination with EBRT in the treatment of lung, head/neck and cervical cancers [11,12]. Furthermore, recent evidence suggests that a positive interaction effect exists also between docetaxel and ADT [13]. All these premises have made docetaxel a suitable agent to integrate into multimodal treatment paradigms for high-risk PCa.

Few studies have reported on the safe use and efficacy of docetaxel at a weekly dose of 20 mg/m^2^ in combination with conventional EBRT in hormone-naïve or ablated high-risk PCa [14-16]. Very little, however, is known about the effect of docetaxel administered concurrently with state-of-the-art high-dose EBRT, e.g. enabled by intensity-modulated radiation therapy (IMRT), and long-term ADT.

In a phase II trial, we evaluated the safety and efficacy of a novel multimodal treatment paradigm for high-risk PCa including high-dose IMRT preceded or not by RP with concurrent intensified-dose docetaxel-based chemotherapy and long-term ADT.

## Methods

### Study population

Eligible subjects were men aged ≥18 years with histologically confirmed PCa and high-risk localized (PSA level ≥20 ng/ml or clinical stage T2c or Gleason score ≥8) or locally advanced (clinical stage T3 or N+, any PSA level, any Gleason score) disease. All patients had abdominal computed tomography, chest X-ray and bone scan excluding distant metastases. Exclusion criteria were: Eastern Cooperative Oncology Group performance status >1; hematological abnormalities (absolute neutrophil count <1500/mm^3^, hemoglobin <8.0 g/dl, platelets <10^5^/mm^3^); liver function tests abnormalities (total bilirubin >1.2 mg/dl, alanine and aspartate transaminase >1.5× the upper normal limit, alkaline phosphatase >2.5× the upper normal limit); renal function tests abnormalities (creatinine >1.5× the upper normal limit); myocardial infarction or unstable angina within 1 year prior to study entry; congestive heart failure (New York Heart Association Class ≥2); uncontrolled chronic disease; severe infection; peripheral neuropathy; prior malignancy (except for non-melanoma skin cancer) within the last 5 years before study entry; prior pelvic external beam radiation therapy; prior chemotherapy; prior prostate surgery; prior treatment for prostate cancer.

The protocol was approved by the local ethics committee of the National Cancer Institute Aviano, and the study was conducted according to the Declaration of Helsinki and European Good Clinical Practice requirements. Written informed consent was obtained at study entry.

### Study design and treatments

Patients were offered one of two multimodal therapy strategies depending on their age at PCa diagnosis. Patients aged ≤65 years were preferentially offered initial open non-nerve-sparing RP with extended pelvic lymph node dissection (PLND) according to a previously reported, anatomically defined template [17], adjuvant EBRT within 3–6 months postoperatively with concurrent docetaxel and long-term ADT (cohort 1). Older patients were preferentially offered primary EBRT with concurrent docetaxel and long-term ADT (cohort 2).

Surgery was performed at two centres (Padua and Pordenone).

All patients were treated with IMRT in Aviano. A 4- to 6-field technique with 15- to 18-MV photon beams was used. No image-guided radiation therapy (IGRT) was used.

In patients of cohort 1, clinical target volume (CTV) included the area from the level of the caudal remnant of the vas deferens, which was intraoperatively marked with clips, to 10 mm inferior to the vesico-urethral anastomosis, from the posterior aspect of the pubis to the anterior rectal wall, and between the medial aspects of each obturator internus muscle. Planning target volume (PTV) consisted of CTV plus a 5-mm margin in all directions except posteriorly, where a 3-mm margin was set. No radiation to the pelvis was given. A total dose of 70 Gy in 35 fractions was prescribed. Dose constraints for organs at risk were: a) bladder: V65 Gy ≤50%, V40 Gy ≤70%; b) rectum: V65 Gy ≤35%, V40 Gy ≤55%; c) femoral heads: V50 Gy ≤10%.

In patients of cohort 2, CTV_1_ included the pelvic area encompassing external iliac, obturator, internal iliac and lower common iliac lymph node chains bilaterally, from the inferior margin of L5 to the inferior margin of the inferior pubic branch, and CTV_2_ included prostate and seminal vesicles. PTV_1_ consisted of CTV_1_ plus a 10-mm margin in all directions, and PTV_2_ consisted of CTV_2_ plus a 10-mm margin in all directions, except posteriorly, where a 6-mm margin was set. A total dose of 80 Gy in 40 fractions was prescribed in three phases: i) first phase: 26 Gy in 13 fractions to PTV_1_ + PTV_2_; ii) second phase: 30 Gy in 15 fractions to PTV_2_; iii) third phase: 24 Gy in 12 fractions to PTV_1_ + PTV_2_. Dose constraints for organs at risk were: a) bladder: V80 Gy ≤15%, V70 Gy ≤35%, V50 Gy ≤60%; b) rectum: V70 Gy ≤20%, V60 Gy ≤35%, V50 Gy ≤50%; c) femoral heads: V50 Gy ≤5%.

Docetaxel was administered starting on the same week as IMRT in a standard 1-hour intravenous weekly dose (30 mg if body surface area <1.8 m^2^ and 40 mg if body surface area ≥1.8 m^2^) for 7 and 8 weeks in patients of cohort 1 and 2, respectively. Premedication with intravenous methylprednisolone, chlorphenamine and ondansetron was given prior to each docetaxel infusion.

ADT, consisting of an injection of a 3-monthly LHRH-agonist, was started either immediately after surgery in cases of pathologically proven seminal vesicle and/or lymph node invasion or 1 to 2 months before IMRT in patients in cohort 1, and 2 to 3 months before treatment in patients of cohort 2. No antiandrogens were given. ADT was prescribed for 24 months in all patients.

### Follow-up

During concurrent IMRT/docetaxel physical examination, complete blood count and toxicity assessment were obtained weekly. After completion, patients were followed with physical examination, urinalysis, serum PSA, liver and renal function tests 3-monthly for the first 2 years, 6-monthly for the following 3 years, and annually thereafter. Follow-up began either on the date of RP (cohort 1) or the date of IMRT completion (cohort 2), and continued until last observation or death.

### Study endpoints

Primary endpoint was safety. Complications after RP were assigned according to the modified Clavien classification system [18]. Toxicity related to concurrent IMRT/docetaxel was scored according to the National Cancer Institute Common Terminology Criteria for Adverse Events version 3.0 [19]. Baseline gastro-intestinal (GI) and genito-urinary (GU) symptoms before IMRT/docetaxel initiation were investigated. Acute toxicity was defined as occurring during and until 3 months after IMRT/docetaxel, while late toxicity as occurring after 3 months until the time of administration of salvage treatment. Similarly to previous investigators [20], toxicity was defined as increase over baseline value for each symptom. All adverse events occurring in a given patient at any follow-up visit were recorded, but only the highest toxicity grade per patient was counted as event when calculating the toxicity rate. To explore toxicity change with time, the incidence of late toxicity at the last follow-up visit was also reported.

Secondary endpoint was efficacy. Biochemical recurrence was defined as an increase in PSA level above 0.2 ng/ml [5] for patients treated with RP or an increase of 2 ng/ml above nadir [21] for patients treated with primary IMRT. Clinical recurrence was defined as local or regional (pelvic lymph nodes) tumour growth, or distant metastases (including retroperitoneal lymph nodes). Biochemical recurrence-free survival (BRFS) and clinical recurrence-free survival (CRFS) were calculated from the date of RP or IMRT completion to the date of biochemical and clinical recurrence, respectively, or death from any cause.

### Statistical analyses

A Simon’s two-stage design was used to calculate the optimal sample size [22]. The trial was designed to test an acute grade ≥3 toxicity rate of ≤5%, considered as acceptable to design a phase III trial, against an acute grade ≥3 toxicity rate of ≥20%, considered as unacceptable, with an 85% power and a 5% significance level. Stopping rule was grade ≥3 toxicity in ≥4 patients during the first stage (17 patients) or in ≥3 patients in the second stage (18 patients). Interim analyses in the second stage were planned every 6 patients.

Continuous variables were non-normally distributed according to Kolmogorov-Smirnov test, and are reported as median with interquartile range (IQR). All analyses were based on intention to treat. Kaplan-Meier estimates were used to explore BRFS and CRFS. Analyses were performed with SAS Enterprise Guide v4.3 (SAS Institute Inc., Cary, NC, USA).

## Results

Between January 2005 and March 2012, 35 men, 18 in cohort 1 and 17 in cohort 2, were enrolled. All patients had complete baseline and follow-up data and were available for the present analysis. Median age was 64 years (IQR 58-69). Median follow-up was 63 months (IQR 31-79). Details are reported in Table 1.

**Table 1:** Clinical and tumor characteristics of patients with high-risk localized or locally advanced prostate cancer enrolled in our phase II trial

**Variable**		**Cohort 1 (n=18)**	**Cohort 2 (n=17)**
Age, years, median (IQR)		59 (56–63)	69 (67–73)
Serum PSA level, ng/ml, median (IQR)		17 (8–26)	18 (9–28)
Clinical T stage, n	T2	6	5
	T3	12	11
	T4	0	1
Clinical N stage, n	N0	16	15
	N+	2	2
Biopsy Gleason score, n	6	3	2
	3+4	1	2
	4+3	4	7
	8-10	9	6
Pathological T stage*, n	T2c	2	NA
	T3a	3	
	T3b	11	
	T4	2	
Positive lymph node status, n		4	NA
Positive surgical margins, n		8	NA
Pathological Gleason score, n	8	5	NA
	9	13	
Follow up, months, median (IQR)		61 (45–80)	63 (26–77)

*IQR* = interquartile range; *NA* = not applicable; *PSA* = prostate specific antigen; *according to the 2002 TNM classification.

All patients in cohort 1 and 14 in cohort 2 completed IMRT to the prescribed dose. Three patients in cohort 2 received 78 Gy because of non-compliance. Eight and 27 patients received 30 mg and 40 mg docetaxel, respectively. Chemotherapy was interrupted in two patients in cohort 2 on initiation of the 2^nd^ cycle because of grade 3 allergic reaction. All patients completed ADT for the prescribed duration.

### Safety

In the first stage, three patients had grade ≥3 toxicity, hence the trial was continued. In the second stage, two further patients had grade ≥3 toxicity, hence the trial was completed.

Perioperative complications after RP and extended PLND were prolonged (>14 days) lymphorrhea (n = 1) (grade I “d”), pelvic hematoma (n = 1) and urinary tract infection (n = 2) (both grade II).

Before IMRT/docetaxel, GI symptoms were absent, while GU symptoms were present in 11 patients, five in cohort 1 and six in cohort 2.

Acute adverse events were common, but most of grade ≤2. All grade 3 events resolved by the time of the subsequent chemotherapy cycle. No grade 4 adverse events were observed. GI and GU symptoms prevailed. Acute GI and GU toxicity was grade 2 in 23% and 20% of patients, and grade 3 in 9% and 3% of patients, respectively. Acute blood/bone marrow toxicity was grade 2 in 20% of patients. All details are reported in Tables 2,3,4,5,6,7.

**Table 2:** Acute adverse events recorded during concurrent high-dose intensity-modulated radiation therapy and docetaxel-based chemotherapy: allergy/immunology

	**Grade**	**Allergic reactions**	**Highest**
Cohort 1	1	1	**1**
	2	0	**0**
	3	0	**0**
Cohort 2	1	0	**0**
	2	1	**1**
	3	2	**2**

**Table 3:** Acute adverse events recorded during concurrent high-dose intensity-modulated radiation therapy and docetaxel-based chemotherapy: blood/bone marrow

	**Grade**	**Anemia**	**Leucopenia**	**Neutropenia**	**Thrombocytopenia**	**Highest**
Cohort 1	1	5	6	3	1	**6**
	2	1	3	0	0	**3**
	3	0	1	0	0	**1**
Cohort 2	1	6	5	2	1	**5**
	2	2	4	1	0	**4**
	3	0	0	0	0	**0**

**Table 4:** Acute adverse events recorded during concurrent high-dose intensity-modulated radiation therapy and docetaxel-based chemotherapy: constitutional symptoms

	**Grade**	**Fatigue**	**Fever**	**Hot flashes**	**Highest**
Cohort 1	1	2	2	2	**4**
	2	1	0	2	**2**
	3	0	0	0	**0**
Cohort 2	1	1	1	2	**3**
	2	2	1	1	**3**
	3	0	0	0	**0**

**Table 5:** Acute adverse events recorded during concurrent high-dose intensity-modulated radiation therapy and docetaxel-based chemotherapy: dermatology/skin

	**Grade**	**Rash/dermatitis**	**Highest**
Cohort 1	1	2	**2**
	2	0	**0**
	3	0	**0**
Cohort 2	1	1	**1**
	2	0	**0**
	3	0	**0**

**Table 6:** Acute adverse events recorded during concurrent high-dose intensity-modulated radiation therapy and docetaxel-based chemotherapy: gastrointestinal symptoms

	**Grade**	**Nausea/vomiting**	**Diarrhea**	**Gastritis**	**Proctitis**	**Hematochezia**	**Highest**
Cohort 1	1	3	8	2	2	2	**8**
	2	2	3	0	1	0	**3**
	3	0	1	0	1	0	**2**
Cohort 2	1	2	7	2	2	1	**7**
	2	3	4	1	1	1	**5**
	3	0	1	0	0	0	**1**

**Table 7:** Acute adverse events recorded during concurrent high-dose intensity-modulated radiation therapy and docetaxel-based chemotherapy: genitourinary symptoms

	**Grade**	**Frequency/urgency**			**Dysuria**			**Incontinence**			**Retention**			**Hematuria**			**Highest**	
		**B**	**A**	A*	**B**	**A**	A*	**B**	**A**	A*	**B**	**A**	A*	**B**	**A**	A*	**A**	A*
Cohort 1	1	3	6	3	1	2	1	3	4	1	0	2	2	0	1	1	**9**	**5**
	2	1	3	2	0	0	0	3	4	1	0	0	0	0	0	0	**7**	**3**
	3	0	0	0	0	0	0	0	0	0	0	0	0	0	0	0	**0**	**0**
Cohort 2	1	3	7	4	2	2	1	0	2	2	3	5	2	0	2	2	**8**	**6**
	2	3	5	2	0	1	1	0	1	1	2	3	1	0	1	1	**8**	**4**
	3	0	0	0	0	0	0	0	0	0	0	1	1	0	0	0	**1**	**1**

*B* = baseline (before concurrent intensity-modulated radiation therapy and docetaxel-based chemotherapy); *A* = all acute adverse events; A* = acute adverse events of higher grade than baseline.

The rate of late adverse events was low and no grade ≥3 toxicity was observed. Late GI and GU toxicity was grade 2 in 9% of patients each. At the last follow-up visit, grade 2 GI and GU toxicity was present in 0% and 6% of patients. No patients underwent surgery for bladder outlet obstruction or urethral stricture. Details are reported in Tables 8,9,10.

**Table 8:** Late adverse events recorded after concurrent high-dose intensity-modulated radiation therapy and docetaxel-based chemotherapy: constitutional symptoms

	**Grade**	**Fatigue**	**Hot flashes**	**Highest during follow-up**	**Highest at last follow-up**
Cohort 1	1	1	3	**3**	**2**
	2	1	0	**1**	**1**
	3	0	0	**0**	**0**
Cohort 2	1	2	2	**3**	**2**
	2	1	1	**1**	**0**
	3	0	0	**0**	**0**

**Table 9:** Late adverse events recorded after concurrent high-dose intensity-modulated radiation therapy and docetaxel-based chemotherapy: gastrointestinal symptoms

	**Grade**	**Diarrhea**	**Gastritis**	**Proctitis**	**Hematochezia**	**Highest during follow-up**	**Highest at last follow-up**
Cohort 1	1	1	1	1	1	**3**	**1**
	2	0	0	1	1	**1**	**0**
	3	0	0	0	0	**0**	**0**
Cohort 2	1	1	0	2	2	**3**	**2**
	2	1	0	1	0	**2**	**0**
	3	0	0	0	0	**0**	**0**

**Table 10:** Late adverse events recorded after concurrent high-dose intensity-modulated radiation therapy and docetaxel-based chemotherapy: genitourinary symptoms

	**Grade**	**Frequency/urgency**		**Dysuria**		**Incontinence**		**Retention**		**Hematuria**		**Highest during follow-up**		**Highest at last follow-up**	
		**L**	L*	**L**	L*	**L**	L*	**L**	L*	**L**	L*	**L**	L*	**L**	L*
Cohort 1	1	3	1	1	0	2	1	0	0	1	1	**5**	**3**	**2**	**1**
	2	0	1	0	0	2	1	0	0	1	1	**2**	**1**	**2**	**1**
	3	0	0	0	0	0	0	0	0	0	0	**0**	**0**	**0**	**0**
Cohort 2	1	3	1	1	0	1	1	2	1	1	1	**6**	**3**	**4**	**2**
	2	2	1	0	0	1	1	2	1	1	1	**3**	**2**	**2**	**1**
	3	0	0	0	0	0	0	0	0	0	0	**0**	**0**	**0**	**0**

*L* = all late adverse events; L* = adverse events of higher grade than baseline.

### Efficacy

One month after RP 12 patients had an undetectable (<0.01 ng/ml) PSA level. Three months after IMRT/docetaxel completion, all patients of cohort 1 had an undetectable PSA level, and all patients of cohort 2 achieved a post-treatment PSA level of ≤1 ng/ml, including 9 patients who achieved an undetectable level.

**Figure 1:**
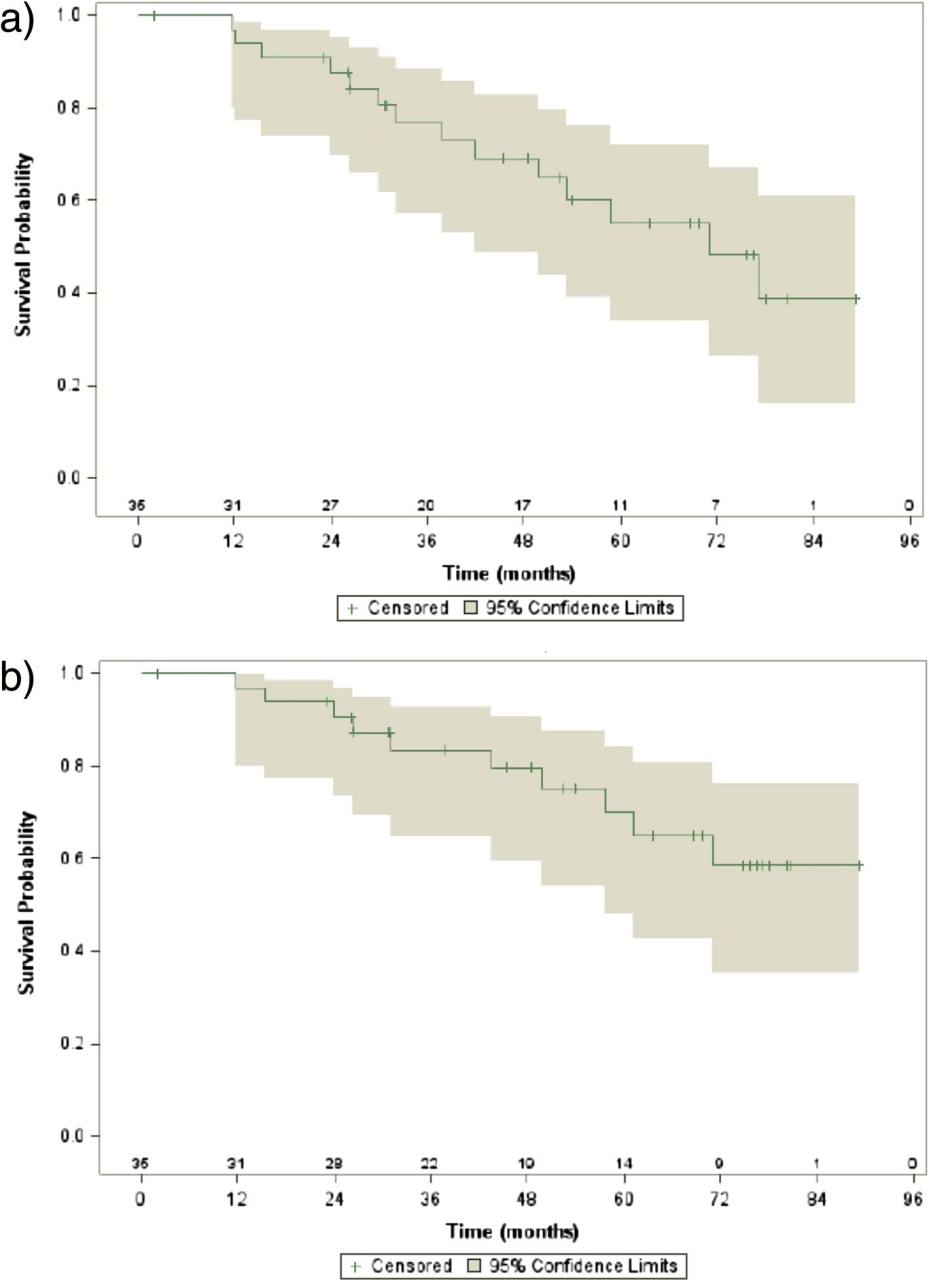
Kaplan-Meier estimates of a) biochemical recurrence-free survival and b) clinical recurrence-free survival.

Fourteen patients (40%), eight in cohort 1 and six in cohort 2, had biochemical recurrence. Actuarial 5-year BRFS rate was 55% (95% CI, 35-75%), 46% (95% CI, 19-73%) for cohort 1 and 68% (95% CI, 42-95%) for cohort 2 (Figure 1a).

Ten patients (29%), five in each cohort, had clinical recurrence. Local recurrence, regional recurrence and distant metastases were detected in three, two and three patients, respectively. The two remaining patients had concomitant local and regional recurrence, and regional recurrence and distant metastases. Actuarial 5-year CRFS rate was 70% (95% CI, 52-88%), 74% (95% CI, 51-96%) for cohort 1 and 65% (95% CI, 36-94%) for cohort 2 (Figure 1b).

Salvage treatment was high-intensity focused ultrasound prostate ablation (n = 1), helical tomotherapy (n = 2), ADT (n = 7), chemotherapy (n = 3), or supportive care (n = 1). Four patients (all in cohort 1) died, two of PCa and two of cardiovascular disease in absence of disease recurrence and ADT.

## Discussion

In the present study, we explored safety and efficacy of a novel multimodal treatment regimen for high-risk PCa based on high-dose IMRT preceded or not by RP, concurrent intensified-dose docetaxel and long-term ADT. Acute toxicity was common but mostly of mild to moderate severity, late toxicity rate was low with no severe adverse events, and mid-term oncological outcome was good.

Six trials have explored the role of concurrent EBRT and taxane-based chemotherapy in patients with high-risk PCa [14-16,23-25] (Table 11). Direct comparison between these and the present trial is hampered by differences in study design, type of EBRT and chemotherapeutic agent used, ADT duration, toxicity assessment and follow-up length. Notably, all previous studies but one [25] applied conventional-dose EBRT only (using either three-dimensional conformal radiation therapy [3D-CRT] or IMRT), which is now considered suboptimal. A meta-analysis, in fact, concluded that high-dose EBRT is associated with a significant improvement in BRFS compared to conventional-dose EBRT, regardless of PCa risk category [26]. Additionally, IMRT has been shown to be able to deliver a higher dose to the prostate while reducing GI and GU toxicity compared to 3D-CRT [27], thus it is currently regarded the most effective form of EBRT.

**Table 11:** Prospective clinical trials evaluating concurrent external beam radiation therapy and taxane-based chemotherapy for high-risk prostate cancer

**Study**	**Design**	**N patients**	**EBRT technique**	**EBRT dosage (Gy)**	**Taxane drug**	**Taxane dosage (mg/m**^**2**^**)**	**ADT duration (months)**	**Toxicity scoring system**	**Highest acute GU toxicity §**	**Highest acute GI toxicity §**	**Highest late toxicity §**	**Biochemical recurrence**	**Follow-up, median (months)**
Kumar [14]	Phase I	22	3D-CRT	70.2	Docetaxel	5 (n=3), 8 (n=3),	None	CTC v2.0, RTOG †	Grade 2	Grade 3	Urinary	5/8	8
						12 (n=3), 16 (n=5),			Frequency/urgency	Diarrhea (n=2)	Retention (n=1)		
						20 (n=6), 25(n=2)			(n=8)				
Sanfilippo [23]	Phase I/II	22	3D-CRT	63 (n=3), 66.6	Paclitaxel	30	9	CTC v2.0	Grade 2	Grade 3	Grade 1	6/22	38
				(n=7), 70.2 (n=4),					Frequency/urgency	Diarrhea (n=4)	Frequency		
				73.8 (n=8)					(n=4)		(n=2)		
Perrotti [15]	Phase I/II	20	IMRT	72	Docetaxel	20	None	CTC,	Grade 2	Grade 2	none	3/20	11.7
								RTOG †	Frequency (n=7)	Diarrhea (n=8)			
Bolla [16]	Phase II	50	3D-CRT (n=45),	70	Docetaxel	20	<36 (n=6),	CTC v2.0,	Grade 3	Grade 4	Grade 3	NR ‡	54
			IMRT (n=5)				36 (n=43),	RTOG †	Dysuria (n=2)	Proctitis (n=1)	Proctitis (n=1)		
							>36 (n=1)						
Hussain [24]	Phase I	59	3D-CRT	70.2 (n=29),	Paclitaxel	40 (n=10),	4 (n=29),	CTC v2.0	Grade 2	Grade 3	NA	13/29, 11/30*	76.3, 74.9*
				64.8 (n=30)*		50 (n=31),	24 (n=30)		Frequency/urgency/	Diarrhea (n=9)			
						60 (n=18)			Incontinence (n=5)				
Chen [25]	Phase I	18	IMRT	78	Docetaxel	10 (n=9),	24	CTCAE v3.0	Grade 2	Grade 3	NA	3/18	26
						15 (n=6),			Frequency (n=2)	Diarrhea (n=2)			
						20 (n=3)							
Present series	Phase II	35	IMRT	80 (n=17),	Docetaxel	30 mg (n=8),	24	CTCAE v3.0	Grade 3	Grade 3	Grade 2 urinary	6/17, 8/18*	63
				70 (n=18)*		40 mg (n=27)			Urinary retention	Diarrhea	Retention		
									(n=1)	(n=2)	(n=2)		

*3D-CRT* = three-dimensional conformal radiation therapy; *ADT* = androgen deprivation therapy; *CTC* = Common Toxicity Criteria of the National Cancer Institute; *CTCAE* = Common Terminology Criteria for Adverse Events of the National Cancer Institute; *EBRT* = external beam radiation therapy; *GI* = gastrointestinal symptoms; *GU* = genitourinary symptoms; *IMRT* = intensity-modulated radiation therapy; *NA* = not assessed; *NR* = not reported; *RTOG* = Radiation Therapy Oncology Group/European Organization for Research and Treatment of Cancer toxicity criteria.

*Patients with previous radical prostatectomy; † late toxicity; ‡ clinical disease-free survival was 66.72% at 5 years; § when two or more events, only the most common was reported.

The only available trial in which high-dose IMRT was used [25] was a phase I trial, where 18 patients with high-risk PCa were treated with high-dose IMRT, concurrent docetaxel at increasing doses and long-term ADT. Only three patients received a dose of 20 mg/m^2^ and none experienced ≥ grade 3 adverse events. In this context, our phase II trial, which used high-dose IMRT, has particular value. In our study, toxicity was common, but was mostly grade ≤2. Most symptoms were of the GI and GU tract. A strength of our analysis is the detailed reporting of both acute and late toxicity, which included the evaluation of baseline symptoms.

Our study included also high-risk PCa patients with prior RP, who may exhibit a different acute and late toxicity profile from their surgery-naïve counterparts, in spite of the lower radiation dose delivered. To the best of our knowledge, only one study [24] explored a similar treatment paradigm also in RP patients. Fifty-nine men with high-risk PCa, including 30 who had undergone RP, were treated with 3D-CRT and concurrent weekly paclitaxel. Although the trial was not designed to test differences between post-RP and non-post-RP patients, overall grade 3 GI and GU toxicity was more common in post-RP patients, whereas estimates of biochemical and clinical recurrence were similar. Our study is different because we used a more modern treatment regimen including high-dose IMRT and docetaxel, but was similarly not equipped for this scope, although both functional and oncological outcome were apparently comparable in the two cohorts.

Over the past few years, there has been an increasing interest towards higher-dose taxane-based chemotherapy. In the ongoing phase III Radiation Therapy Oncology Group-0521 trial (ClinicalTrials.gov, NCT00288080), which is evaluating whether adjuvant docetaxel to EBRT with ADT provides a survival advantage over EBRT with ADT alone in high-risk localized PCa, a high dosage of 75 mg/m^2^ every 3 weeks for 6 weeks is used. In our study, an intensified dosage of docetaxel was used compared to previous trials. This was decided in an attempt to further improve the oncological outcome, given the tolerability of the 20 mg/m^2^ dose established in the pivotal trial by Kumar et al. [14]. The above-mentioned limitations notwithstanding, toxicity profile in the present and previous trials is apparently comparable.

Concerning the oncological outcome, our results are comparable to those of the two trials with the longest follow-up, where a 41% biochemical recurrence rate [25] and a 67% 5-year CRFS [16] were observed. One may argue that our mid-term biochemical outcome is similar to that reported in previous studies using conventional-dose EBRT with long-term ADT [28]. Nonetheless, we believe that our results do justify the conduct of a phase III trial where high-risk patients are randomized between high-dose IMRT with ADT plus concurrent docetaxel and high-dose IMRT with ADT alone, with appropriate oncological outcomes as the primary endpoint. In fact, our treatment paradigm might prove beneficial if hard endpoints (e.g. metastasis-free and cancer-free survival) and a sufficiently long-term follow-up are considered. On the other hand, a recent study on ultra-high-dose (86.4 Gy) IMRT for localized PCa [29] has reported higher BRFS rates (67.9%) in high-risk patients at a similar follow-up (5.5 years). One may speculate that IMRT delivered at this very high dose can achieve a better improvement, at least, in local control, than docetaxel-based chemo-radiation. However, whether concurrent docetaxel confers a true benefit has to be seen in the pattern of clinical recurrence, where the incidence of regional and distant metastases is expected to be lower. Future research in this area is eagerly awaited.

To the best of our knowledge, two phase II/III trials and one phase III trial testing EBRT with ADT plus concurrent docetaxel vs EBRT with ADT alone in high-risk PCa are currently recruiting patients (ClinicalTrials.gov, NCT00116142, NCT01603420 and NCT01811810). However, in none of these trials the experimental arm entails a combination of high-dose IMRT, intensified-dosage docetaxel-based chemotherapy and long-term ADT, such as in our trial.

Our study is not devoid of limitations. First, no control group was available. Second, toxicity was graded according to a physician-based assessment and health-related quality of life was not assessed. Third, IGRT may have contributed to improve the toxicity profile. Fourth, the choice between RP and RT was based on an arbitrary age cut-off with its inherent limitations. Fifth, as per our trial protocol post-RP EBRT was given to patients with either extracapsular disease, seminal vesicle invasion or positive surgical margins, which are all established risk factors for local recurrence. The latest results of a large randomized trial on adjuvant EBRT vs observation following RP for high-risk PCa [28] showed that patients with positive surgical margins benefit most from adjuvant EBRT in subgroup analyses. Our study started in 2005, i.e. at a time when mature data on post-RP EBRT outcomes were not available. However, even currently, the impact of positive surgical margins on biochemical and clinical recurrence remains under debate. In another similar randomized trial [30], surgical margin status was not an independent predictor of biochemical outcome. Finally, irradiation to pelvic lymph nodes in addition to the prostate in cN0 high-risk PCa, as in our cohort 2 patients, remains controversial. One option would be a staging PLND, whereby pN0 patients might be spared irradiation and pN1 patients might benefit from irradiation with long-term ADT [5].

## Conclusions

In a phase II trial including patients with high-risk localized or locally advanced PCa we showed acceptably low toxicity and good efficacy of a multimodal treatment including high-dose IMRT preceded or not by RP, concurrent intensified-dose docetaxel and long-term ADT. Thus, this treatment paradigm may warrant further evaluation in phase III randomized trials.

## Abbreviations

3D-CRT: Three-dimensional conformal radiation therapy; ADT: Androgen deprivation therapy; BRFS: Biochemical recurrence-free survival; CRFS: Clinical recurrence-free survival; CTV: Clinical target volume; EBRT: External beam radiation therapy; GI: Gastro-intestinal; GU: Genito-urinary; IGRT: Image-guided radiation therapy; IMRT: Intensity-modulated radiation therapy; IQR: Interquartile range; PCa: Prostate cancer; PLND: Pelvic lymph node dissection; PTV: Planning target volume; RP: Radical prostatectomy.

## Competing interests

The authors declare that they have no competing interests.

## Authors’ contributions

RB and FiZ: study conception and design. AGu, FaZ, MG and VP: acquisition of data. AGu, GG, PG, AGa, RB and FiZ: analysis and interpretation of data. AGu, GG and PG: drafting of the manuscript. AGu and FiZ: surgery. RB: radiation therapy planning. FV: statistical analyses. RB and FiZ: critical revision of the manuscript for important intellectual content, and supervision. All authors read and approved the final manuscript.
